# The INTREST registry: protocol of a multicenter prospective cohort study of predictors of women’s response to integrative breast cancer treatment

**DOI:** 10.1186/s12885-021-08468-2

**Published:** 2021-06-23

**Authors:** Heidemarie Haller, Petra Voiß, Holger Cramer, Anna Paul, Mattea Reinisch, Sebastian Appelbaum, Gustav Dobos, Georg Sauer, Sherko Kümmel, Thomas Ostermann

**Affiliations:** 1 Department of Internal and Integrative Medicine, Evang. Kliniken Essen-Mitte, Faculty of Medicine, University of Duisburg-Essen, Am Deimelsberg 34a, 45276 Essen, Germany; 2 Breast Unit, Evang. Kliniken Essen-Mitte, Essen, Germany; 3 Department of Psychology, Chair of Research Methodology and Statistics in Psychology, Witten / Herdecke University, Witten, Germany; 4 Department of Gynecology and Obstetrics, Robert-Bosch-Hospital, Stuttgart, Germany

**Keywords:** Breast Cancer, Predictors, Treatment response, Integrative Cancer treatment, Complementary medicine

## Abstract

**Background:**

Cancer registries usually assess data of conventional treatments and/or patient survival. Beyond that, little is known about the influence of other predictors of treatment response related to the use of complementary therapies (CM) and lifestyle factors affecting patients’ quality and quantity of life.

**Methods:**

INTREST is a prospective cohort study collecting register data at multiple German certified cancer centers, which provide individualized, integrative, in- and outpatient breast cancer care. Patient-reported outcomes and clinical cancer data of anticipated *N* = 715 women with pTNM stage I-III breast cancer are collected using standardized case report forms at the time of diagnosis, after completing neo−/adjuvant chemotherapy, after completing adjuvant therapy (with the exception of endocrine therapy) as well as 1, 2, 5, and 10 years after baseline. Endpoints for multivariable prediction models are quality of life, fatigue, treatment adherence, and progression-based outcomes/survival. Predictors include the study center, sociodemographic characteristics, histologic cancer and comorbidity data, performance status, stress perception, depression, anxiety, sleep quality, spirituality, social support, physical activity, diet behavior, type of conventional treatments, use of and belief in CM treatments, and participation in a clinical trial. Safety is recorded following the Common Terminology Criteria for Adverse Events.

**Discussion:**

This trial is currently recruiting participants. Future analyses will allow to identify predictors of short- and long-term response to integrative breast cancer treatment in women, which, in turn, may improve cancer care as well as quality and quantity of life with cancer.

**Trial registration:**

German Clinical Trial Register DRKS00014852. Retrospectively registered at July 4th, 2018.

**Supplementary Information:**

The online version contains supplementary material available at 10.1186/s12885-021-08468-2.

## Background

With an estimated prevalence of 100.5 million in 2017, cancer remains one of the leading causes of mortality worldwide [1]. For patients diagnosed with breast cancer, the prognosis has continuously improved, resulting in 33.8% more years lived with disability over the last decade [1]. Thus, aspects of quality of life and management of side effects during and after cancer treatment have progressively increasing impact. Common symptoms associated with the diagnosis and treatment of cancer include fatigue, sleep disturbances, affective disorders, pain and neuropathy, reported by more than half of all patients [2–4]. In order to improve the quality of life, cancer patients often use complementary medicine (CM) [5–8]. On average, up to 40% of cancer patients use CM across Europe [6], with breast cancer patients being the largest group [6, 9]. They, however, tend not to disclose their usage of CM treatments to their treating oncologists [6]. In the worst case, this lack of communication can lead to severe interactions between complementary and conventional therapies that can have a negative impact not only on quality but also on quantity of life [10–13].

In contrast, integrative oncology “is a patient-centered, evidence-informed field of cancer care that utilizes mind and body practices, natural products, and/or lifestyle modifications from different traditions alongside conventional cancer treatments. Integrative oncology aims to optimize health, quality of life, and clinical outcomes across the cancer care continuum and to empower people to prevent cancer and become active participants before, during, and beyond cancer treatment” [14]. The concept is based on the NIH definition of integrative health care [15] and Clinical Practice Guidelines of the Society for Integrative Oncology (SIO) [16], endorsed by the American Society of Clinical Oncology (ASCO) [17]. A systematic review published in 2012 identified 29 integrative cancer programs, situated in the United States, England, Canada, and Germany [18], all of them established between 1968 and 2007. In 2010, in Germany another program was launched that provides integrative oncology for breast cancer patients by incorporating evidence-based complementary therapies into standard in- and outpatient cancer care [19]. Beside conventional curative and adjuvant cancer treatments and supportive psycho-oncological care, the individualized CM treatments offered in this program include:

CM treatments administrated by physicians/naturopaths/acupuncturists such as

- individual and group-based educative consultations about the benefits and risks of CM therapies in the management of side effects of conventional cancer treatment,
- natural products such as herbs, dietary supplements, mistletoe, and other anthroposophical/homeopathic remedies,
- ear and body acupuncture and acupressure, neural therapy, gua sha therapy (traditional Chinese skin scraping massage), and cupping,

CM treatments administrated by mind-body medicine therapists such as

- individual and group-based educative consultations about the benefits and risks of lifestyle modifications in the areas of exercise, nutrition, relaxation, cognitive restructuring, and naturopathic coping strategies,
- courses in yoga, tai chi and qi gong, art therapy, mindfulness, relaxation, and meditation,

CM treatments administrated by nurses such as

- aromatherapy with and without (rhythmical) massage, therapeutic foot massage, footbaths, compresses and poultices, teas and mouthwashes,
- psychosocial support groups [19–21].

To address requested CM therapies with currently insufficient scientific evidence, a traffic light system has been developed [21]. Therapies are given a green light and can be used if they are of low risk and are known to have valuable clinical effects. Promising therapies that are of higher risk as well as those that have shown a limited extent of efficacy while having only low risks are given a yellow light and might be used in selected cases. Therapies with limited effectiveness and high risks shall not be applied (red light).

Randomized controlled trials have shown preliminary comparative effectiveness of integrative cancer programs in contrast to treatment as usual. One trial investigated outpatients with breast and gynecologic cancer undergoing chemotherapy and found that an integrative complex nursing intervention over 24 weeks customized to the patients’ symptomatic burden and preferences improved aspects of quality of life and fatigue [22]. Another trial examined breast cancer patients in different treatment phases who were randomized to either standard care alone or standard care integrating individualized CM treatments provided by trained physicians and nurses during a period of 26 weeks. Analyses revealed significant between-group differences on cancer-related quality of life, pain, fatigue, and function in favor of the integrative therapy [23]. However, RCT designs often do not include follow-ups longer than 6 or 12 months, which limits their external validity. Clinical and epidemiological cancer registries, on the other hand, follow patients without time restrictions but usually do not go further than assessing data on conventional treatment algorithms and patient survival.

Predictors of response to cancer treatment/patient survival include, in particular, tumor characteristics and access to innovative, timely screening and treatment strategies [24–27]. Moreover, factors of resilience related to mental, physical and social variables, such as the patient’s ability to adapt to psychological distress or stressful life events without developing mental health disorders [28, 29], exercise and healthy diet [30–32], are shown to be predictors of survival from breast cancer. A registry assessing data on the influence of both conventional and CM treatment and of mental and physical resilience using qualitative and quantitative variables of treatment response has not been established yet.

The INTREST registry aims to identify independent predictors of treatment-response in women undergoing individualized, integrative breast cancer treatment combining conventional as well as supportive complementary therapies. Treatment response is defined in terms of quality of life, fatigue, treatment adherence to conventional cancer care, and progression-based variables.

## Methods

### Study setting and design

We developed the INTREST registry as an epidemiological, prospective, multi-center cohort design according to the STROBE and TRIPOD guidelines [33, 34]. The study protocol was reported according to the SPIRIT guideline [35]. Each study site was approved by the respective ethics committee and registered at the WHO International Clinical Trials Registry Platform/German Clinical Trials Register (DRKS00014852). The 2017 version of the INTREST protocol comprised two certified cancer centers in Germany, one in Essen (Breast Unit, Evang. Kliniken Essen-Mitte) and a second in Stuttgart (Department of Gynecology, Robert-Bosch-Hospital). The 2019 version includes a third center located in Münster, Germany (Breast Unit, St. Franziskus-Hospital). Involving further study sites is within the scope of the protocol.

Qualified study sites have to be certified cancer centers providing conventional breast cancer diagnosis and treatment procedures as well as integrate in- and/or outpatient complementary medical visits, treatment offers, and nursing interventions [19, 21]. Women shall be treated by an interdisciplinary medical team of breast cancer specialists (radiologists, pathologists, breast surgeons, and oncologists), complementary physicians, psycho-oncologists, mind-body therapists, or breast care nurses trained in complementary medicine. CM treatments are offered in accordance with the individual indication and preference during and after active cancer therapy up to 10 years post diagnosis.

**Fig. 1:**
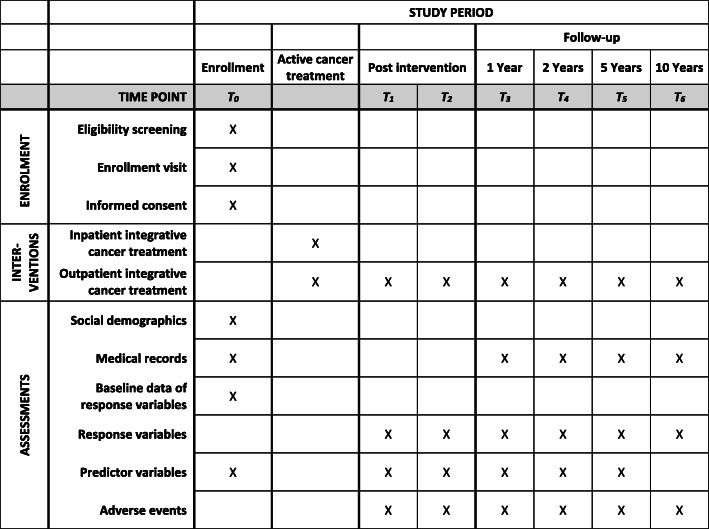
Study flow diagram

Measurement points include a baseline assessment at the time of diagnosis (T0), two individual time points after completing the neo−/adjuvant chemotherapy (T1) and/or after finishing all adjuvant treatments (except for endocrine therapy) (T2), a one-year follow-up (T3) as well as further consultations at year two (T4), year five (T5), and year ten (T6). In cases of withdrawal of consent or death, study participation ends earlier. A summary of the study design can be found in Fig. 1.

### Eligibility criteria

Patients are eligible, if they are female, have been diagnosed with primary breast cancer stage I-III (according to the pTNM classification) [36], and receive individualized cancer treatments in one of the participating study centers. Breast cancer diagnosis has to be confirmed by histological classification following breast biopsy according to the currently applicable guidelines [37, 38]. Exclusion criteria are male sex, hospitalization due to treatment of a benign tumor, carcinoma in situ, relapse or metastases. Women who report suffering from a severe comorbid somatic disease or being under psychopharmacological treatment for a psychiatric or neurodegenerative disorder, such as dementia, major depression or a psychosis that make them unable to participate in the study are excluded, as well as those who do not have the sufficient knowledge of the German language.

### Recruiting procedure

Eligible women are selected from tumor board lists of the participating hospitals that contain the latest on-site histologic, genetic, and clinical diagnoses as well as treatment recommendations. Study nurses inform eligible patients about the study procedure and provide written study information as part of the tumor board outpatient visit, where women receive their individualized treatment plans. In order to be included in the study, interested women have to give their written informed consent and fill out the baseline questionnaire. Reasons for non-participation are recorded.

Follow-up questionnaires are delivered by mail at pre-defined time points and are monitored by reminder calls. Replacement questionnaires are provided when necessary [39]. In cases of no response, additional questionnaires are sent via email. Considering the 10-year follow-up period and possible mental deterioration, a screening tool for cognitive impairment is included. The Mail-In Cognitive Function Screening Instrument (MCFSI) is assessed at T5 and T6 [40]. In cases of ≥5 points on the MCFSI [41], respective questionnaires are checked for response bias and excluded from analyses/further monitoring if necessary. In general, study staff is instructed to verify survival status of each woman by checking the internal cancer registry before sending the respective questionnaire.

### Data collection

#### Outcomes of treatment response

Endpoints for the multivariate prediction model include patient reported outcomes (PROs), treatment adherence, and progression-based outcomes.

PROs include quality of life and fatigue, which are assessed by the Functional Assessment of Cancer Therapy General (FACT-G) [42] and the associated Fatigue Scale (FACIT-F) [43]. The FACT-G total score ranges from 0 to 108 points. The highest possible score for the physical, social, and functional well-being sub scales is 28, and 24 for the emotional well-being sub scale. The FACIT-F ranges from 0 to 52 points. Both questionnaires have been well validated and have shown good reliability [44, 45] as well as a better responsiveness for the detection of clinically relevant changes compared to the FACT-B [46] or the EORTC QLQ-C30 [47].

Treatment responders at the time points T1 to T2 are defined as women who will score above the 25th percentile of the female US adult population (> 68 points on the FACT-G and > 33 points on the FACIT-F) [44] and will reach the same or higher scores in comparison to their respective baseline values at T0. Non-responder are defined as women who will score below the respective 25th percentile of the female US adult population or will report a minimal clinically important worsening (7 points on the FACT-G total score or 4 points on the FACIT-F) [45, 48] in comparison to their respective baseline values at T0. Responders at the time points T3 to T6 are defined as women who will score above the 50th percentile of the female US adult population (> 83 points on the FACT-G and > 42 points on the FACIT-F) [44] and will reach a minimal clinically important improvement (> 5 points on the FACT-G total score or 4 points on the FACIT-F) [44, 45] in comparison to their respective baseline values at T0. Non-responders are defined as women who will score below the respective 50th percentile or will report a minimal clinically important worsening.

Adherence to chemotherapy and endocrine therapy is assessed at T1 and T4, T5, and T6, respectively. Patients who do not adhere to chemo- or endocrine therapy due to adverse events are defined as non-responders, others as responders.

Progression-based outcomes recommended for the adjuvant setting [49] include Invasive Disease-Free Survival (IDFS), Distant Relapse-free Survival (DRFS), Breast Cancer Specific Survival (BCSS) and Overall Survival (OV). Treatment response and non-response for all progression-based outcomes is assessed at T3 to T6.

#### Predictors

Predictor variables include sociodemographic characteristics at T0 such as age, race, marital status, kind of health insurance, education, occupation, height and weight, lifestyle habits (alcohol intake, smoking behavior, physical activity, and diet), characteristics of menstruation, pregnancy and the use of hormonal contraception, other hormonal drugs for in vitro fertilization, abortion or menopausal symptoms, and radiation exposure. Patients are also questioned about their beliefs about CM therapies using the 10-item CAM Health Belief Questionnaire (CHBQ) [50], and about their prior use of and interest in CM using a 10-point numeric rating scales (NRS).

Further clinical data at T0 are obtained from medical records. Those include time of diagnosis, histology, previous oncological disease, hereditary risk score, pTNM and if indicated ypTNM, grading, hormone receptor status, menopausal status, ECOG and Karnofsky performance status, and physical and mental comorbidity according to the 35-item Health-Related Quality of Life Comorbidity Index (HRQL-CI) [51].

Predictors assessed by questionnaires at all time points include stress perception using the 10-item Questionnaire on Distress in Cancer Patients Short Form (QSC-R10) [52], depression using the 10-item Center for Epidemiologic Studies Depression Scale Short Form (CESD-SF10) [53, 54], hopelessness using the negatively worded 2-item Brief Hopelessness measure (BH-NEG) [55], state anxiety using the Patient-Reported Outcomes Measurement Information System Emotional Distress Anxiety 4-item Short Form (PROMIS-EDA-SF4) [56], progression anxiety using the 5-item Fear of Relapse/Recurrence Scale (FRRS) [57], emotion regulation using the 4-item expressive suppression subscale of the Emotion Regulation Questionnaire (ERQ) [58, 59], sleep disturbance using the Patient-Reported Outcomes Measurement Information System Sleep Disturbance 4-item Short Form (PROMIS-SD-SF4) [60], spiritual well-being using the 12-item Functional Assessment of Cancer Therapy Spiritual Well-Being Scale (FACIT-SP) [61], social support using the 8-item perceived available support subscales of the Berlin Social Support Scales (BSSS) [62], physical activity using the 4-item International Physical Activity Questionnaire (IPAQ) [63], and diet using the 14-item Mediterranean Diet Adherence Screener (MEDAS) [64].

Finally, we assess variables of the study center and the need for and type of conventional treatment procedures including data on the surgical intervention, chemotherapy, radiotherapy, and endocrine therapy as well as whether or not the patient is participating in a clinical trial. At all follow-ups, the patients are asked about their use of complementary therapies (provided at the study center as well as used by patients themselves) by an extended version (59 predefined and additional free-text items instead of originally 29 ones) of the International Complementary and Alternative Medicine Questionnaire (I-CAM-Q) [65–67]. Adverse events are recorded by the 32-item Short Form of the Memorial Symptom Assessment Scale (MSAS-SF) [68] following the Common Terminology Criteria for Adverse Events. Satisfaction with the treatments is assessed by the 8-item Client Satisfaction Questionnaire (CSQ) [69].

### Data management and quality assurance

All patient-reported data and those collected from medical records are transferred into standardized paper CRFs by trained medical staff of the respective study site. After formal checking for completeness, data from the paper CRFs are entered into a specially designed online database based on WINDOWs package XAMPP including an Apache Server, a MySQL-Database, and PHP as the dialect of the framework [70].

The INTREST-database uses a web-application, which serves as a secured user interface to access the database. Entered data is validated during the data entry process using programmed validation checks concerning item types, checks for required values and item ranges. For each discrepancy, a “discrepancy note” is stored in the system and resolved by cross-checking the entries in the database with the source data. Discrepancies that cannot be solved, are indicated as “unsolved” in the system and will be discussed at a regular data review meeting.

The INTREST-registry is provided to each study site and administrated centrally by the evaluating body (Prof. Dr. Thomas Ostermann, University of Witten / Herdecke). Transfer of registry data takes place exclusively in a pseudonymized form by the study centers. Thus, the conclusion on an individual participant is not possible. For statistical analysis, registry data are transferred into a predefined csv-file format. Also at this stage, data are examined for accuracy and completeness by random comparisons between the csv-file and the original database.

### Statistics

#### Sample size estimation

Taking into account k independent variables as covariates and a defined ratio p of responders to non-responders at the respective follow-up points, a required number of cases of *N* = 10 k/p are needed [71]. To avoid overfitting and in line with the recommendations given by Vittinghoff & McCulloch, 2007 [72] we assume to arrive at ten predictors out of the above mentioned variables. Together with a ratio p of 1:5 of responders to non-responders this results in a sample size of *N* = 10 × 10/0.20 = 500. To compensate for power losses due to drop-outs of up to 30%, for T0, a sample size of *N* = 715 is calculated.

#### Dealing with missing data

In responder analysis, there are two strategies to deal with missing data: imputation of missing data prior to dichotomization or dichotomization prior to imputation. In simulation studies, imputing data before dichotomization was shown to be less biased compared with imputing the dichotomous response [73]. Therefore, data missing at random will be imputed i.e. by using Markov chain Monte Carlo methods.

#### Statistical analysis plan

The method to be used for predicting responders and non-responders will be based on imputation strategies for responder analysis given in the model selection approach by Schomaker & Heumann, 2014 [74]. In practical terms, we follow the outline given in Rethorst et al., 2017 [75]. The single steps in our approach are displayed in the supplementary material and described as follows:

From the original set of data, a set of bootstrap samples *B*_*i*_ *(i = 1 … m)* is drawn. Missing values in each bootstrap sample are replaced by multiple imputation techniques described above resulting in *k* completed data *B*_*ij*_ *(i = 1 … m; j = 1,k)* sets for each bootstrap sample. For each of the data sets *B*_*ij*_ a separate model selection approach is carried out using two different methods.

1. A logistic regression model using a least absolute shrinkage and selection operator (LASSO) approach [76]: Although similar to the classical logistic regression model, the LASSO regression model has an additional penalty term λ > 0, which shrinks coefficients of predictors with low predictive power towards zero or exactly to zero [77].
2. A random forest (RF) classification method: the random forest is a classification method based on the binary classification and regression trees (CART) method in data mining, first introduced by Breiman, 2001 [78]. Based on k learning sets, the K decision trees form a random forest. Then, the majority vote of these trees is used to make an ensemble classification decision [79].

Resulting predictors P_*ijl*_ of these modeling approaches will be pooled and the k estimates for each bootstrap sample *B*_*i*_ will be averaged. Based on these results, a final model for each selection approach will be created taking into account the rank and relative strength of the predictors from each bootstrap sample.

The final two models (one for the LASSO and one for the RF approach) are used for a) a classical logistic regression model and b) a Classification and Regression Trees (CART) approach. In this final evaluation step, a validation of the predictors is performed using the compete cases of the out of back samples O_i_ *(i = 1 … m)* left over from the bootstrap datasets. All statistical analyses are going to be run with R.

## Discussion

The analysis of the INTREST registry data will reveal predictors of response to integrative breast cancer treatment. Challenges of the study design may include the overall large number of questionnaires as well as the 10-year follow-up, which may result in a high number of drop-outs. To improve patient initial participation and attendance, only significant predictors will remain within the revised INTREST-registry, while non-significant variables/questionnaires will be removed. In addition, comprehensive monitoring strategies shall ensure higher patient compliance.

For other cancer centers providing integrative cancer care, the adapted INTREST-registry may provide an interesting platform to answer questions of health services research. Selected significant predictors might also be of interest for standard clinical cancer registries to extend their validity and reliability. Particularly, if the use of CM therapies proves to be a significant predictor, those with adequate evidence of effectiveness and safety may be suggested to be integrated into standard cancer care. This will serve the needs of the women diagnosed with breast cancer [8, 9, 80] and may improve quality of care for cancer as well as quality and quantity of life with cancer.

### Trial status

The trial is currently recruiting participants.

## Supplementary Information

**Additional file 1.** Statistical analysis plan.

## Data Availability

The dataset analyzed during the study is not publicly available as it include personal/pseudonymized information. Fully anonymized data will be available from the corresponding author on reasonable request.

## Abbreviations

ASCO: American Society of Clinical Oncology
BCSS: Breast Cancer Specific Survival
CAM: Complementary and Alternative Medicine
CART: Classification and Regression Trees
CM: Complementary Medicine
CRF: Case Report Form
CSF: Comma-separated values (Excel) file
DRFS: Distant Relapse-free Survival
ECOG: Eastern Cooperative Oncology Group performance status
IDFS: Invasive Disease-Free Survival
INTREST: RESposne to INTegrative breast cancer Treatment
Λ: Lambda
LASSO: Least absolute shrinkage and selection operator
MCFSI: Mail-In Cognitive Function Screening Instrument
MYSQL: Open-source relational database management system
OV: Overall Survival
PHP: Programming language
PRO: Patient Reported Outcome
pTNM: Classification of Malignant Tumors, assessed after surgery
R: Free programming language software
RF: Random Forest
SIO: Society for Integrative Oncology
STROBE: STrengthening the Reporting of OBservational studies in Epidemiology guideline
T: Time point of data assessment
TRIPOD: Transparent Reporting of a multivariable prediction model for Individual Prognosis Or Diagnosis guideline
XAMPP: Open-source web server solution package by Apache Friends
yTNM: Classification of Malignant Tumors, assessed after neoadjuvant chemotherapy and/or radiation therapy
