# Supplementary material for: The INTREST registry: protocol of a multicenter prospective cohort study of predictors of women’s response to integrative breast cancer treatment
Source: BMC Cancer. 2021 Jun 23;21:724. doi: 10.1186/s12885-021-08468-2 (PMC8220783; doi:10.1186/s12885-021-08468-2)
Supplement: Supplementary file 1 — Additional file 1. Statistical analysis plan. [file 12885_2021_8468_MOESM1_ESM.docx]

Final prediction model

Accuracy of the model (i.e. ROC-Analysis,…)

Complete cases from the out of the back samples (O_1_, P_2_, …O_m_)

Validation of the Predictors P_i_

Averaging and final selection of the Predictors P_i_

Final Set of predictors (P_1_, P_2_, P_3_,…P_r_)

P_m-11_P_m-12_…
P_m-1n_

_0_

…

…

P_21_P_22_…
P_2n_

P_11_P_12_…
P_1n_

P_m1_P_m2_…
P_mn_

P_mk1_P_mk2_…
P_mkn_

P_m21_P_m22_…
P_m2n_

P_m11_P_m12_…
P_m1n_

…

Pooling of the Predictors P_ijl_

Model selection resulting in Predictors P_ijl_

P_1k1_P_1k2_…
P_1kn_

P_121_P_122_…
P_12n_

P_111_P_112_…
P_11n_

…

Multiple Imputation

…

B_11_

B_12_

B_1k_

B_1_

B_2_

B_m_

B_m-1_

Bootstrapping

Original data
